# Ventilatory Management of Patients with Acute Respiratory Distress Syndrome Due to SARS-CoV-2

**DOI:** 10.3390/jcm12247509

**Published:** 2023-12-05

**Authors:** Marine Jacquier, Marie Labruyère, Fiona Ecarnot, Jean-Baptiste Roudaut, Pascal Andreu, Pierre Voizeux, Quentin Save, Romain Pedri, Jean-Philippe Rigaud, Jean-Pierre Quenot

**Affiliations:** 1 Department of Intensive Care, François Mitterrand, University Hospital, 21000 Dijon, France; marine.jacquier@chu-dijon.fr (M.J.); marie.labruyere@chu-dijon.fr (M.L.); jean-baptiste.roudaut@chu-dijon.fr (J.-B.R.); pascal.andreu@chu-dijon.fr (P.A.); pierre.voizeux@chu-dijon.fr (P.V.); quentin.save@chu-dijon.fr (Q.S.); romain.pedri@chu-dijon.fr (R.P.); 2 Lipness Team, INSERM Research Centre LNC-UMR1231 and LabEx LipSTIC, University of Burgundy, 21000 Dijon, France; 3 INSERM CIC 1432, Clinical Epidemiology, University of Burgundy, 21000 Dijon, France; 4 Department of Cardiology, University Hospital Besancon, 25030 Besançon, France; fiona.ecarnot@univ-fcomte.fr; 5 EA3920, University of Franche-Comté, 25000 Besançon, France; 6 Department of Intensive Care, Centre Hospitalier de Dieppe, 76202 Dieppe, France; jrigaud@ch-dieppe.fr; 7 Espace de Réflexion Éthique de Normandie, University Hospital Caen, 14000 Caen, France; 8 DRCI, USMR, CHU Dijon Bourgogne, 21000 Dijon, France; 9 Espace de Réflexion Éthique Bourgogne Franche-Comté (EREBFC), University of Burgundy, 21000 Dijon, France

**Keywords:** management, acute respiratory distress syndrome, COVID-19, ICU, SARS-CoV-2

## Abstract

The emergence of the new SARS-CoV-2 in December 2019 caused a worldwide pandemic of the resultant disease, COVID-19. There was a massive surge in admissions to intensive care units (ICU), notably of patients with hypoxaemic acute respiratory failure. In these patients, optimal oxygen therapy was crucial. In this article, we discuss tracheal intubation to provide mechanical ventilation in patients with hypoxaemic acute respiratory failure due to SARS-CoV-2. We first describe the pathophysiology of respiratory anomalies leading to acute respiratory distress syndrome (ARDS) due to infection with SARS-CoV-2, and then briefly review management, focusing particularly on the ventilation strategy. Overall, the ventilatory management of ARDS due to SARS-CoV-2 infection is largely the same as that applied in ARDS from other causes, and lung-protective ventilation is recommended. The difference lies in the initial clinical presentation, with profound hypoxaemia often observed concomitantly with near-normal pulmonary compliance.

## 1. Introduction

The pandemic caused by SARS-CoV-2 resulted in a sudden and massive surge in the numbers of patients being admitted to intensive care units (ICUs) with hypoxaemic acute respiratory failure [1,2]. For these patients, ensuring optimal oxygen therapy was crucial. Despite conflicting findings reported to date [3,4,5], it appears reasonable in view of the current state of knowledge to propose oxygen therapy at a level that achieves arterial oxygen saturation (SaO_2_) between 92 and 98%. This target makes it possible to avoid severe hypoxaemia while limiting the risks of hyperoxaemia and hyperoxia [6]. To achieve this target, intensivists have several techniques at their disposal, such as high-concentration oxygen masks, high-flow nasal cannula [7], continuous positive airway pressure [8], non-invasive ventilation [9] or mechanical ventilation after orotracheal intubation [2]. Here, we discuss mechanical ventilation only, since the other techniques will be dealt with in separate contributions. Since the COVID-19 pandemic, as with hypoxaemic acute respiratory failure of other causes, the question of the timing of intubation and the criteria that qualify a patient for intubation remain hotly debated [10]. Indeed, performing intubation too early incurs a risk of futile intubations, exposing patients to the possible risk of complications associated with invasive ventilation [11]. Conversely, delayed intubation incurs a twofold risk, namely the risk of hypoxic cardiac arrest and the risk of “patient self-inflicted lung injury” (PSILI), whereby there may be an aggravation of pulmonary injury due to the mechanical stress on the lung by prolonged and intense breathing efforts [12]. In addition, during the early waves of the COVID-19 pandemic, the limited amount of resources compared to the disproportionately high number of patients underscored the importance of careful evaluation of patients to avoid non-beneficial intubations [13]. It should be noted that the very severe hypoxaemia observed in some patients with COVID-19 has been dubbed “happy hypoxia” by some authors, reflecting the fact that it was not the main driver of dyspnoea and polypnea during acute respiratory failure [14]. Accordingly, the course of changing oxygen parameters and/or the presence of clinical signs of respiratory failure were considered as more important contributors than hypoxaemia alone to the decision to intubate. Indeed, early data in the COVID-19 literature reported variable rates of use of invasive mechanical ventilation, ranging from 42% in China [15] to 71% in the United States [16], 80% in France [2] and 88% in Italy [1]. In the same way, mortality in the ICU and in-hospital also varied widely (from 25% to 90%) and depended largely on the severity of patients at admission to the ICU, socio-economic factors, associated comorbidities and the quality of the country’s healthcare system [1,2,17,18]. In this work, we describe the pathophysiology of respiratory anomalies leading to acute respiratory distress syndrome (ARDS) due to infection with SARS-CoV-2, its management, focusing particularly on the ventilation strategy, and associated treatments.

## 2. Pathophysiology of ARDS Due to SARS-CoV-2

ARDS can be the direct result of primary epithelial injury (for example, bacterial or viral pneumonia) [19]. The insult leads to activation of alveolar macrophages, initiating a pulmonary inflammatory response, which attracts inflammatory cells and causes pulmonary oedema via an increase in capillary permeability and destruction of the alveolar-capillary barrier. Typical histological lesions show diffuse alveolar damage.

During SARS-CoV-2 infection, the virus penetrates cells via the angiotensin converting enzyme. In some patients, there ensues a phase of pulmonary infection affecting the pneumocytes in the alveolar–capillary barrier. This in turn leads to cell death through apoptosis and piroptosis, as well as via macrophage activation and a dysregulated immune response [20]. As in other forms of ARDS, the consequences of this process include fibrin-rich interstitial and alveolar oedema, as well as endothelial and epithelial injury (destruction of the alveolar–capillary barrier), leading to leakage mediated by fibroblasts, with production of collagen and a healing process that can progress to pulmonary fibrosis. These inflammatory processes cause a loss of aerated lung volume, a concept dubbed “baby lung”. In the immediate term, this can result in profound hypoxaemia with a reduction in pulmonary compliance due to increases in elastic recoil, intra-pulmonary shunt and dead space [12].

The most recent definition of ARDS, known as the Berlin definition, was proposed by a panel of experts convened as an initiative of the European Society of Intensive Care Medicine (ESICM) endorsed by the American Thoracic Society and the Society of Critical Care Medicine and was first published in 2012 [21]. Three forms of ARDS can be distinguished [22], namely focal ARDS, requiring PEEP generally lower than 10 cm H_2_O and which responds well to prone positioning [23]; diffuse ARDS, which tends to respond better to high PEEP (>10 cm H_2_O) and, finally, mixed ARDS.

More recently, a consensus conference of 32 critical care ARDS experts suggested expanding the Berlin ARDS definition due to the various developments in treatment, including (amongst others) the use of high-flow nasal oxygen (HFNO) [24]. HFNO is used to treat hypoxaemic respiratory insufficiency, as in the FLORALI study [7], and its use became very widespread during the COVID-19 pandemic [25,26,27]. Indeed, the original Berlin definition only includes patients requiring non-invasive and/or invasive techniques for the treatment of acute respiratory insufficiency, and the definition is poorly applicable in limited-resource settings [28], especially when blood gas analysis is not available [29]. The expert committee therefore made four updated recommendations [24], namely (1) include HFNO with a minimum flow rate of ≥30 L/min; (2) use arterial oxygen tension (PaO_2_)/FiO_2_ ≤ 300 mmHg or SpO_2_/FiO_2_ < 315 (if SpO_2_ ≤ 97%) to identify hypoxaemia; (3) retain bilateral opacities for imaging criteria but add ultrasound as an imaging modality, especially in resource-limited areas; and (4) in resource-limited settings, do not require PEEP, oxygen flow rate or specific respiratory support devices.

In the case of lung injury due to SARS-CoV-2, the time interval between the first respiratory symptoms and the onset of ARDS is around 7 to 10 days [30]. In terms of the mechanics of ventilation, there is a conspicuous absence of pulmonary compliance abnormalities, despite the presence of hypoxaemia that can be as severe as in ARDS cases typically found in intensive care [31]. Gattinoni et al. [32] classified ARDS into two categories according to lung compliance, namely “L-type” profiles (low elastance), or type 1, with near-normal compliance; and “H-type” (high elastance), or type 2, with decreased compliance. In the L-type, the near-normal compliance indicates that the amount of non-aerated lung tissue is low, and that recruitability must therefore be poor. In the H-type, pulmonary compliance is decreased, with substantial loss of aerated lung tissue, which theoretically means that there is potentially high recruitability. Computed tomography (CT) shows alveolar condensation associated with interstitial infiltrate. Both profiles (types 1 and 2) can be observed during the course of the disease, and the ventilation strategy may contribute to the progression of type 1 towards type 2 ARDS. Similarly, situations where the patient’s breathing is not adapted to the ventilator, such as patients who are breathing spontaneously with high respiratory drive, may contribute to the development of patient self-inflicted lung injury (PSILI) due to an increase in transpulmonary pressure [12].

A further specificity of SARS-CoV-2 pneumonia is the presence of micro- and macro-thrombus in the lungs, which could play a key role in the pathogenesis of infection in the context of endothelial disease [33]. Indeed, arterial and venous thromboembolic events occur in up to 30% of critically ill COVID-19 patients [34]. Other, rarer disorders have also been described, such as antiphospholipid syndrome or thrombotic microangiopathy [35]. In any case, the occurrence of pulmonary embolism can exacerbate hypoxia and complicate the ventilation conditions, notably due to pulmonary arterial hypertension. When there is right heart failure induced by pulmonary embolism, hypoxaemia can be further exacerbated by a PvO_2_ effect caused by the drop in cardiac output, which leads to oxygen desaturation of venous blood. These data suggest that ARDS caused by SARS-CoV-2 infection could be associated with a more marked increase in dead space, as compared to “conventional” ARDS [36,37]. Beyond the right heart dysfunction caused by massive pulmonary embolism, COVID-19 patients more frequently present right systolic and diastolic dysfunction. Potential hypotheses to explain this include mechanical ventilation and hypercapnia [38,39]. This right heart dysfunction was more frequently described with COVID-19 than in other forms of ARDS, whereas left heart dysfunction, likely due to septic cardiomyopathy, was observed at the same frequency as in other causes of ARDS. Finally, dysregulation of pulmonary vasomotricity also appears to be implicated. Indeed, a mismatch is observed between the severe hypoxaemia, the relative absence of dyspnoea, and the preserved lung compliance (with conserved aerated lung). Taken together, these findings suggest that the hypoxaemia is the reflection of altered hypoxic pulmonary vasoconstriction and dysregulated blood flow, which cause a reduction in the ventilation/perfusion ratio [40].

## 3. Ventilatory Management of Respiratory Failure Caused by SARS-CoV-2 Infection

An expert consensus statement was published in 2021 detailing the recommendations for the management of respiratory failure caused by SARS-CoV-2 infection [41]. The authors recommend considering the use of invasive mechanical ventilation when one or more of the following criteria are present: altered mental status; hemodynamic instability; failure to maintain SpO_2_ > 90% with other non-invasive respiratory techniques; persistent respiratory distress; a subjectively observed increase in the work of breathing; or PaO_2_/FiO_2_ < 100. Conversely, tachypnea (defined as a respiratory rate ≥ 30/min) and PaO_2_/FiO_2_ < 200 were not retained by the expert consensus group as criteria for initiation of tracheal intubation.

In the acute phase of the disease, a so-called “lung-protective” strategy is most frequently employed, using tidal volumes of 4–7 mL/kg of predicted body weight, and a target plateau pressure ≤ 30 cm of H_2_O and driving pressure (plateau pressure—total PEEP) ≤ 15 cm of H_2_O are recommended with a view to limiting ventilator-induced lung injury [41,42]. It may also be necessary to tolerate possible hypercapnia and respiratory acidosis, as long as they remain relatively mild [43].

Despite these general recommendations, there is a likely a need to personalize the ventilation strategy in each patient, taking into account their specific physiological characteristics and needs. This is particularly true as regards the level of PEEP. Indeed, although, as expected, PEEP improves oxygen uptake during COVID-related ARDS, this effect is not always due to significant alveolar recruitment. In some patients, it may be partially, if not entirely, due to the hemodynamic consequences of PEEP (i.e., the relationship between cardiac output and intrapulmonary shunt) [44]. It should be noted that the expert consensus statement does not recommend recruitment manoeuvres, optimizing PEEP according to FiO_2_, monitoring of transpulmonary pressure with an oesophageal balloon or use of pressure–volume curves [41]. Accordingly, evaluation of the effects of PEEP as compared to blood gas (PaO_2_ and PaCO_2_) and mechanical findings (driving pressure and compliance) should make it possible to personalize the ventilation settings. As mentioned above, Gattinoni et al. classified ARDS into two categories according to lung compliance, namely “L-type” profiles (low elastance), or type 1, with near-normal compliance; and “H-type” (high elastance), or type 2, with decreased compliance [32]. This difference in compliance could be explained by the levels of angiotensin 2 and ICAM, which are higher in patients with normal compliance [45,46]. This underlines the major role of the endothelium, although further prospective investigations are warranted to allow definitive conclusions to be drawn. Thanks to the description of the L and H types proposed by Gattinoni [32], it may also be possible to identify patients who will or will not respond to an increase in PEEP. Thus, patients with an H profile could likely be ventilated with higher PEEP. Conversely, it is advisable to limit tidal volume and maintain a driving pressure < 14 cm of H_2_O in order to keep plateau pressure below 30 cm of H_2_O. In a study of 17 intubated and mechanically ventilated subjects with COVID-19 ARDS, respiratory system compliance decreased significantly when increasing PEEP, without a significant variation in the PaO_2_/FiO_2_ ratio [47]. Patients with lower compliance had an increased shunt, suggesting that increased PEEP could be deleterious for these patients. This is in line with discussions about the overstretching of recruitable zones and thus the measure of compliance in the recruitable tissue, for example, using the r/I ratio or emerging techniques for measuring impedance.

**Figure 1:**
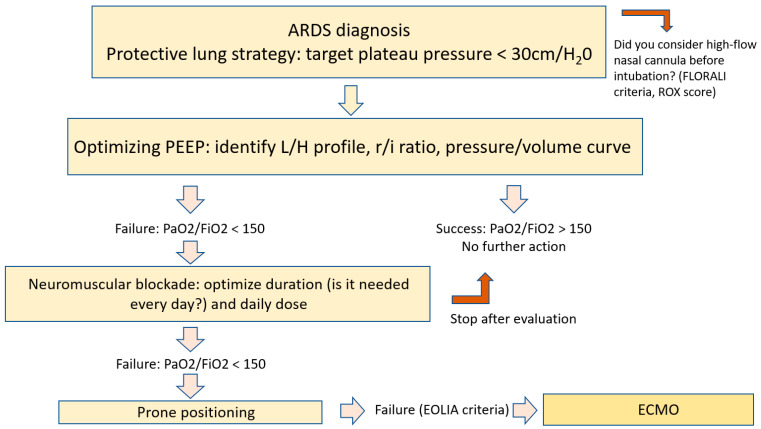
Proposed algorithm for a personalized ventilation strategy. ARDS, acute respiratory distress syndrome; L/H, low/high elastance; r/i ratio, recruitment to inflation ratio; ECMO, extracorporeal membrane oxygenation.

The so-called “protective” strategy raises several questions: First, how can we evaluate the optimal ventilatory strategy likely to limit damage induced by mechanical ventilation? Second, how can we evaluate optimal tidal volume, according to predicted weight, the measure of driving pressure or other approaches? Third, how can we personalize ventilation based on individual physiological data? We propose an algorithm for a personalized ventilation strategy in Figure 1. Finally, could we evaluate other approaches to reducing PaCO_2_ induced by the use of low tidal volumes, which may aggravate lesions induced by mechanical ventilation?

The management of patients with COVID-related ARDS has raised numerous ethical dilemmas because of the saturation of hospitals at epidemic peaks. This led to an increased awareness of considerations such as recommendations, patient values and preferences, access to and use of available resources, the acceptability of management, especially in the ICU, the feasibility of implementing highly technical care and also equity in healthcare [48].

The 2017 guidelines for mechanical ventilation in ARDS [49] were updated recently [50] and point out the mismatch between the conceptual model of ARDS (a specific form of inflammation and host response to a lesion [21]), and the absence of any measure of inflammation in the definitions of ARDS. Identifying a particular sub-type of ARDS is undoubtedly important for the personalization of management, as was shown in the LIVE study [51]. Indeed, in that study, Constantin et al. randomly assigned 420 patients to either standard “protective” ventilation (control group) or a strategy personalized based on the radiological phenotype (focal or non-focal ARDS on chest CT or X-ray). The personalized strategy was not shown to reduce mortality at 90 days. However, as the authors explain, misclassification of over 20% of patients, leading to treatment attribution errors, could be responsible for the failure to observe a significant effect. Of note, post hoc analysis on the per-protocol population (i.e., only patients whose lung morphology was correctly classified) showed a benefit in favour of the personalized ventilation strategy. In the future, in the setting of prospective randomized studies, it will likely be necessary to perform real-time tests at the bedside to evaluate the phenotype. These tests will need to be quick and operator-independent, pending the advent of specific biomarkers of pulmonary injury, such as those used in cardiac disease, kidney disease and sepsis.

In patients with acute hypoxaemia respiratory distress that is not due to cardiogenic pulmonary oedema or to exacerbation of COPD, it is recommended to use HFNO rather than conventional oxygen therapy, in order to mitigate the risk of intubation and need for mechanical ventilation. This recommendation is also applicable for patients presenting with COVID-19. Indeed, conventional oxygen therapy using a face mask or nasal cannula is limited by its low oxygen flow (less than 15 L/min) and the absence of humidification of the inspired oxygen, which can give rise to patient complaints. Conversely, HFNO is well tolerated, delivering heated, humidified oxygen at flows up to 60 L/min [52]. Similarly, HFNO can deliver a more constant inspiratory fraction of oxygen (FiO_2_) than conventional oxygen therapy, while also reducing the dead space and providing PEEP of up to 3–5 cm H_2_O, depending on the flow and how the patient breathes [53]. Although the use of HFNO now appears to be consensually accepted in patients with hypoxaemic respiratory insufficiency, a number of questions remain unanswered. For example, it is not yet clear whether preventing intubation can mitigate respiratory symptoms and improve the long-term functional capacity of the lungs in survivors. Similarly, it remains to be established how long HFNO should ideally be used for and on which criteria to base a decision to intubate.

In this regard, the use of scores such as the ROX score (defined as the ratio of oxygen saturation/FiO_2_ to respiratory rate) has been investigated, with Roca et al. reporting that a ROX score > 4.88 at 2, 6 or 12 h after HFNC initiation was consistently associated with a lower risk for intubation [54], even though the reasons for intubating were not specifically described. These results were confirmed in a sub-analysis of FLORALI [7]. The ROX score was also retrospectively studied in a cohort of patients with COVID-19, which confirmed that increasing ROX index score at the time of intubation was associated with greater survival [55].

The experts in the consensus conference that updated the ARDS definition [50] were unable to make recommendations regarding the use of HFNO rather than non-invasive ventilation in patients not suffering from cardiogenic pulmonary oedema or COPD, in the absence of conclusive clinical trials in this particular setting [56,57].

When comparing the 2017 recommendations [49] with those of 2023 [50], it is apparent that a “protective” ventilatory strategy is recommended in patients with COVID-19, but no specific recommendation is made regarding the management of PEEP, irrespective of the ARDS aetiology. The recruitability of lung tissue, without inducing overdistension, has been well established and understood by ICU physicians since 2006 [58]. However, techniques for measuring potentially recruitable volumes remain somewhat burdensome to implement, such as electrical impedance tomography [59]. A study by Chen et al. in 2019 investigated a bedside approach for estimating lung recruitability and calculating compliance of the recruited lung, thus optimizing PEEP levels in these patients [60]. Larger, randomized studies are needed to confirm the value of this approach, which has the advantage of being easy to implement at the bedside.

## 4. Neuromuscular Blockade

Since the publication of the ACURASYS study [61], it is now well established that the early use of neuromuscular blocking agents during ARDS reduces barotrauma lesions and ventilator-induced lung injury (VILI), thereby improving survival and reducing the duration of mechanical ventilation, without increasing ICU-acquired paresis. However, in that study, neuromuscular blockade was of short duration (maximum 48 h), so early re-evaluation of this approach seems warranted, at the risk of increasing the risk of myopathy [62]. Monitoring the degree of neuromuscular blockade, for example, with train-of-four (TOF) testing [63], seems to be central to reducing the doses of neuromuscular blocking agents consumed. However, adhering to the recommended 48 h duration of neuromuscular blockade during the COVID-19 pandemic was difficult because of the longer durations of ventilation in these patients [64], and due to difficulties with sedation with probable tachyphylaxis [65], rendering early withdrawal of neuromuscular blocking agents complex. Furthermore, while the use of neuromuscular blocking agents clearly improves oxygenation over the first 48 h of ARDS [66], it did not have any impact on the duration of mechanical ventilation, explaining why the systematic use of neuromuscular blockade is called into question. In this regard, Payen et al. underlined the importance of individual management of sedation and neuromuscular blockade, including in patients infected with SARS-CoV-2, for example, by using multimodal analgesia [67].

## 5. Prone Positioning

Prone positioning is a key component of a lung-protective ventilation strategy, especially in patients with PaO_2_/FiO_2_ < 150 [23]. Prone positioning reduces the incidence of lung injury by reducing atelectasia and derecruitment of the most dependent lung tissue, as observed in the supine position. Indeed, prone position redistributes the mechanical forces at play in the lung, leading to more uniform pulmonary expansion and recruitment of dependent lung regions. Available literature data indicate that the proportion of responders to prone positioning in terms of oxygenation response during ARDS due to SARS-CoV-2 is identical to that observed in non-COVID-related ARDS [68]. A recent study also highlighted the utility of awake prone positioning of patients with hypoxaemic respiratory failure due to COVID-19 to reduce the need for intubation [69].

The consensus conference published in 2023 [50] maintained their opinion regarding prone positioning, including in COVID-19 patients, notably those without invasive ventilatory support (awake prone positioning) [69]. A recent meta-analysis reported improved survival when prone positioning was used in ARDS patients receiving veno-venous ECMO [70]. Indeed, Ehrmann et al. reported in a multicentre, randomized, open-label trial including 1126 patients that awake prone positioning for hypoxaemic respiratory failure due to COVID-19 significantly reduced treatment failure and the need for intubation, without any signs of potential harm, notably in terms of cutaneous lesions due to the prone position [69].

However, other, more recent studies have not replicated these findings [71,72], including in meta-analyses of randomized trials [73,74]. Brunelle et al. evaluated the impact of awake prone positioning on the index of ventilation as measured using electrical impedance tomography in patients with acute respiratory failure due to COVID-19 [75]. They reported improved oxygenation in patients with awake prone positioning, without a decrease in lung ventilation inhomogeneity, as assessed using electrical impedance tomography. The response to first prone position, in terms of a sustained improvement in oxygenation after a first PP session was shown to be independently associated with improved survival and a reduced duration of mechanical ventilation in critically ill COVID-19 patients [76]. However, other reports suggest that only a small proportion of critically ill patients are suitable for awake prone positioning, and those who are eligible cannot tolerate many episodes [77]. Therefore, results regarding awake prone positioning remain to be confirmed by further studies.

The conflicting findings of all these studies in terms of the impact of awake prone positioning on ventilation or prognosis raise a number of questions, notably about the ideal daily duration of prone positioning sessions. Second, what level of hypoxaemia should trigger implementation of prone positioning, and third, how can we maximize patient comfort and promote adherence with this manoeuvre? Indeed, patient comfort seems to be a key issue, as investigated in a qualitative study by Zhu et al. [78] among 19 patients to identify barriers to and facilitators of adherence to awake prone positioning. Among the key facilitators identified by these authors were availability and affordability of treatment, family influences and perceived benefits. Furthermore, promoting positive beliefs and attitudes among patients vis à vis treatment and debunking any misconceptions or fears they harbour could also serve to enhance adherence to treatment. Elsewhere, Godoy et al. investigated the combination of awake prone positioning with HFNO in a systematic review of 13 studies totalling 1242 patients [79]. They concluded that there was an encouraging pathophysiological rationale for the use of this combination of techniques to improve oxygenation, but they suggest that consensus remains warranted regarding the criteria for timing, management and, above all, discontinuation before this approach could be generalized.

## 6. Inhaled Nitric Oxide

Data regarding the effect of inhaled NO on arteria oxygenation are conflicting [80,81]. Data from the multi-centre, prospective COVID-ICU cohort study reported that 425 (19%) of the 2233 patients with ARDS on day 1 received inhaled NO, albeit without the possibility of drawing any formal conclusion regarding the efficacy of inhaled NO in this indication [2]. In a retrospective multicentre study published by Mekontso-Kessap et al., the authors reported a benefit of inhaled nitric oxide for arterial oxygenation in patients with acute ARDS. However, they found no impact in terms of mortality, and concluded that these findings warrant confirmation in prospective, randomized trials.

## 7. Extracorporeal Assistance

Veno-venous extracorporeal membrane oxygenation (ECMO) makes it possible to reduce the ventilatory pressure exerted on the injured lung, thereby potentially mitigating ventilator-induced lung injury in patients with severe disease. In ECMO, blood from the systemic circulation of the patient is directed to the extracorporeal oxygenator, where it is enriched in oxygen via a membrane and carbon dioxide is removed. Then, the blood is reinjected into the patient. ECMO remains an exceptional therapy that should only be proposed for selected patients with persisting profound hypoxaemia despite optimization of ventilatory parameters and the use of prone positioning and in the absence of any pre-existing conditions that jeopardize the patient’s life expectancy in the short to medium term. This position was confirmed by the recent updated recommendations [50]. The criteria for initiation of ECMO are those used in the EOLIA study, namely PaO_2_/FiO_2_ < 60 mmHg for >6 h, PaO_2_/FiO_2_ < 50 mmHg for >3 h or arterial blood pH < 7.25 plus PaCO_2_ ≥ 60 mmHg for >6 h [82]. The current state of knowledge does not justify any change in the indications or management of veno-venous ECMO [41]. The timing of ECMO implantation should be the same as in ARDS from other causes, namely within 7 days after onset of ARDS. The utility of early implantation of ECMO remains debated [83]. Weaning of COVID-19 patients who required veno-venous ECMO remains an open question. This issue was investigated by Masi et al. in a single-centre study that showed a longer duration of ventilation and more frequent need for prone position in patients who were weaned earlier. The respective total durations of mechanical ventilation were 21 days in the conventional ECMO weaning group versus 55 in the facilitative ECMO weaning group. These findings warrant further investigation and confirmation.

Several new anticoagulation strategies have been tested for ECMO in COVID-19 patients, in order to reduce thromboembolic events [84,85], and the haemorrhagic complications of veno-venous ECMO implantation in COVID-19 patients have been described in the literature [86]. However, the indication for anticoagulation, including in COVID-19-related ARDS with a high thromboembolic risk, does not prevent bleeding events, especially intracranial haemorrhage. The use of newer anticoagulants such as bivalirudin calls this paradigm into question [84,85] and appears to achieve a reduction in thrombosis of the ECMO circuit without a need for increased doses. However, existing results are conflicting [87].

## 8. Conclusions

The ventilatory management of ARDS due to SARS-CoV-2 infection is largely the same as that applied in ARDS from other causes, and lung-protective ventilation is recommended. The difference lies in the initial clinical presentation, with profound hypoxaemia often observed concomitantly with near-normal pulmonary compliance. Similarly, the vascular anomalies specific to COVID-19, with a marked alteration of hypoxic pulmonary vasoconstriction, also count among the major differences between COVID-related and non-COVID-related ARDS and warrant further investigation in future studies.

## Data Availability

Not applicable. This paper does not report any data.
